# Evaluation of a Camera-Based Monitoring Solution Against Regulated Medical Devices to Measure Heart Rate, Respiratory Rate, Oxygen Saturation, and Blood Pressure

**DOI:** 10.7759/cureus.31649

**Published:** 2022-11-18

**Authors:** Debjyoti Talukdar, Luis Felipe De Deus, Nikhil Sehgal

**Affiliations:** 1 Medical Research, Mkhitar Gosh Armenian-Russian International University, Yerevan, ARM; 2 AI Research, Vastmindz Limited, London, GBR

**Keywords:** camera-based monitoring, rppg, remote photoplethysmography, medical device, blood pressure, oxygen saturation, respiratory rate, heart rate

## Abstract

Background

Regularly monitoring common physiological signs, including heart rate, blood pressure, and oxygen saturation, can effectively prevent or detect several potential conditions. In particular, cardiovascular diseases (CVDs) are a worldwide concern. According to the World Health Organization, 31% of all deaths worldwide are from CVDs. Recently, the coronavirus disease 2019 pandemic has increased the interest in remote monitoring. At present, contact devices are required to extract most of an individual’s physiological information, which can be inconvenient for users and may cause discomfort.

Methodology

However, remote photoplethysmography (rPPG) technology offers a solution for this issue, enabling contactless monitoring of the blood volume pulse signal using a regular camera. Ultimately, it can provide the same physiological information as a contact device. In this paper, we propose an evaluation of Vastmindz’s rPPG technology against medical devices in a clinical environment with a variety of subjects in a wide range of age, height, weight, and baseline vital signs.

Results

This study confirmed the findings that the contactless technology for the estimation of vitals proposed by Vastmindz was able to estimate heart rate, respiratory rate, and oxygen saturation with a mean error of ±3 units as well as ±10 mmHg for systolic and diastolic blood pressure.

Conclusions

Reported results have shown that Vastmindz’s rPPG technology was able to meet the initial hypothesis and is acceptable for users who want to understand their general health and wellness.

## Introduction

There is a growing interest in technologies related to remote patient monitoring (RPM) solutions, an interest that has largely been piqued amid the coronavirus disease 2019 (COVID-19) pandemic. However, COVID-19 has not only caused several deaths worldwide but has also led to uncountable consequences and side effects. Long before the pandemic, cardiovascular diseases (CVDs) have been impacting the world. According to the World Health Organization (WHO), 17.9 million people die each year from this condition, representing around 31% of deaths worldwide. CVDs are a group of health complications relating to the heart and blood vessels and include coronary heart disease, cerebrovascular disease, rheumatic heart disease, and other conditions [1]. Therefore, it is critical to regularly monitor some physiological parameters which can lead to early detection of such health complications.

Well-established methods for capturing physiological data include the use of the electrocardiogram (ECG) or photoplethysmography (PPG), both of which require the use of contact sensors. These methods, through their physiological signals, have the ability and the potential to measure several different physiological parameters such as heart rate (HR), respiration rate (RR), oxygenation (SpO_2_), and blood pressure (BP). Alternatively, researchers have recently introduced the remote photoplethysmography (rPPG) technique which is a low-cost, non-contact method and an alternative solution for measuring the same parameters as the PPG signal in a contactless way. Because it is a method that can be performed on any consumer technology device with an embedded camera, its ease of use makes it an attractive addition to the suite of RPM solutions.

The information acquired through rPPG reflects the variations of blood volume in skin tissue which is modulated by cardiac activity. The reflection of light is influenced by the change in the volume of blood and of the movement of the wall of blood vessels, which is visible within frame-to-frame changes of a red, green, and blue (RGB) camera. However, there are several challenges to extracting an optimal rPPG signal. Distortion in the signal might be caused by low illumination, significant head movement, and the device properties in low-end devices such as the camera’s frame rate and its resolution.

Any rPPG technology usually relies on a four-step methodology, which can be summarized as frame-to-frame extraction, region of interest (ROI) detection, signal processing, and vitals estimation. First, the video files are usually separated into several frames, the amount of frames in a certain period is denoted as frame rate, measured in frames per second (FPS). There is, however, a minimum FPS required to pick up fast changes in the cardiac cycle. The ROIs are theoretically compared to small sensors placed on the face and can diverge among authors, although a common step would be the detection of landmarks, which is performed by detecting face regions in each video frame. This process is commonly used with face-tracking algorithms such as the Viola-Jones method [2]. Once the ROIs are selected, pixel intensity components are extracted, and those components are in the RGB color space. In addition, the RGB components are spatially averaged over all pixels in the ROI to yield a red, blue, and green component for each frame and form the raw signals.

Furthermore, the signal processing stage is applied, also known as the “rPPG Core.” The rPPG Core has been the subject of various studies in the last decade, resulting in multiple methods that seek to extract the signal as cleanly as possible from RGB components. Some rely on blind source separation (BSS) methods, which can retrieve information by de-mixing raw signals into different sources. Principal component analysis (PCA)-based and independent component analysis (ICA)-based methods use different criteria to separate temporal RGB traces into uncorrelated or independent signal sources are some of the techniques used [3,4]. Other authors have tried to improve the quality of the signal by changing the color space to a chrominance-based domain [5].

In a more recent study, Wang et al. [6] introduced a new alternative to process RGB components into rPPG signal, called the plane-orthogonal-to-skin (POS) algorithm. The algorithm’s main idea is to filter out the intensity variations by projecting RGB components on a plane orthogonal to a determined normalized skin tone vector. As a result, a two-dimensional signal referent to the projections is obtained and then combined into a one-dimensional (1D) signal which is one of the input signal dimensions that is weighted by an alpha parameter. The alpha parameter is the quotient of the standard deviations of each signal. This article was previously posted to the medRxiv preprint server on April 27, 2020.

## Materials and methods

This study aimed to compare the accuracy of remote health screening technology against gold standard vital signs monitors (hereon referred to as the reference method) in the measurement of HR, RR, BP, and SpO_2_. The study involves evaluating the accuracy of HR, RR, BP, and SpO_2_ using Vastmindz’s 3.0 SDK which can be integrated into android and iOS mobile apps as well as Web apps and provides an estimation of physiological assessments for a variety of vital signs. It is worth considering that the reference instruments used also have some degree of error and in some cases can deviate by up to ±3 units per parameter. This will be taken into account when discussing the results.

Methods

**Figure 1:**
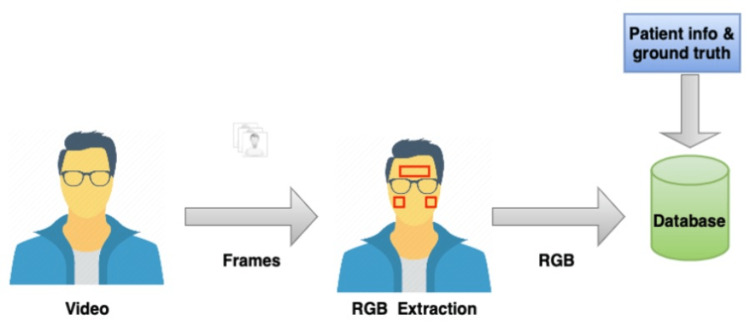
Study methodology: data collection. RGB: red, green, and blue

**Figure 2:**
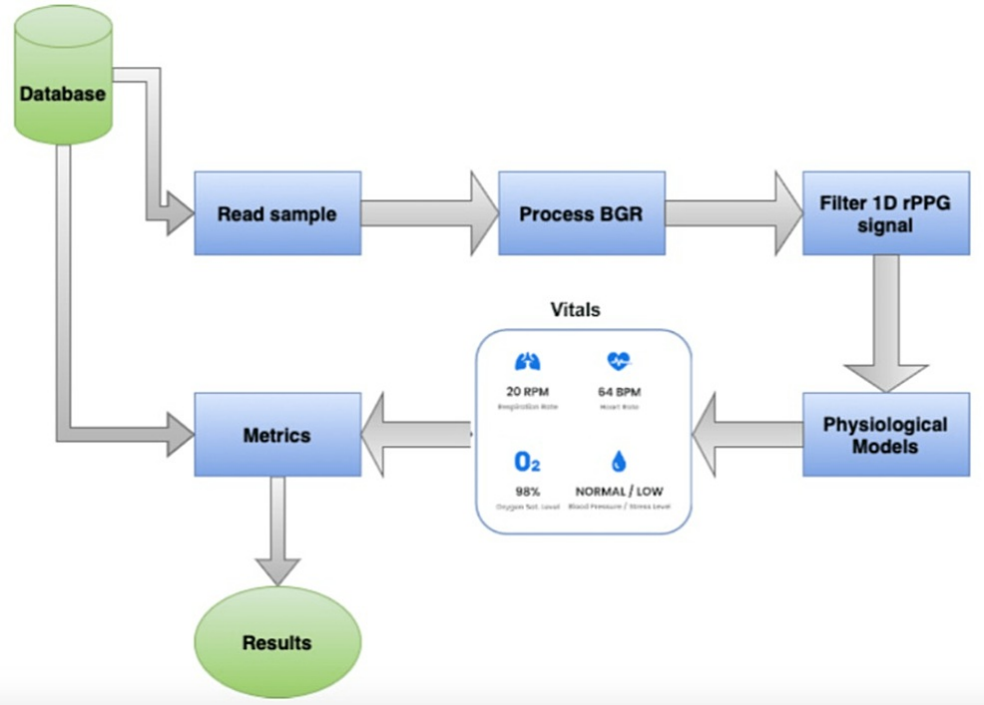
Study methodology: analysis. BGR: blue, green, and red; 1D: one-dimensional; rPPG: remote photoplethysmography

The methods of this study are divided into two blocks, namely, the onsite data collection and the analysis. The data were collected at the hospitals without any interference from any staff. The device used in data collection was running an app with Vastmindz’s technology. The app interface effectively performs face detection, places landmarks, and creates ROIs, after which BGR components of each ROI within each frame are collected and stored in a cloud database. The BGR samples are the only visual information stored. Figure 1 and Figure 2 show an overview of the method.

**Figure 3:**
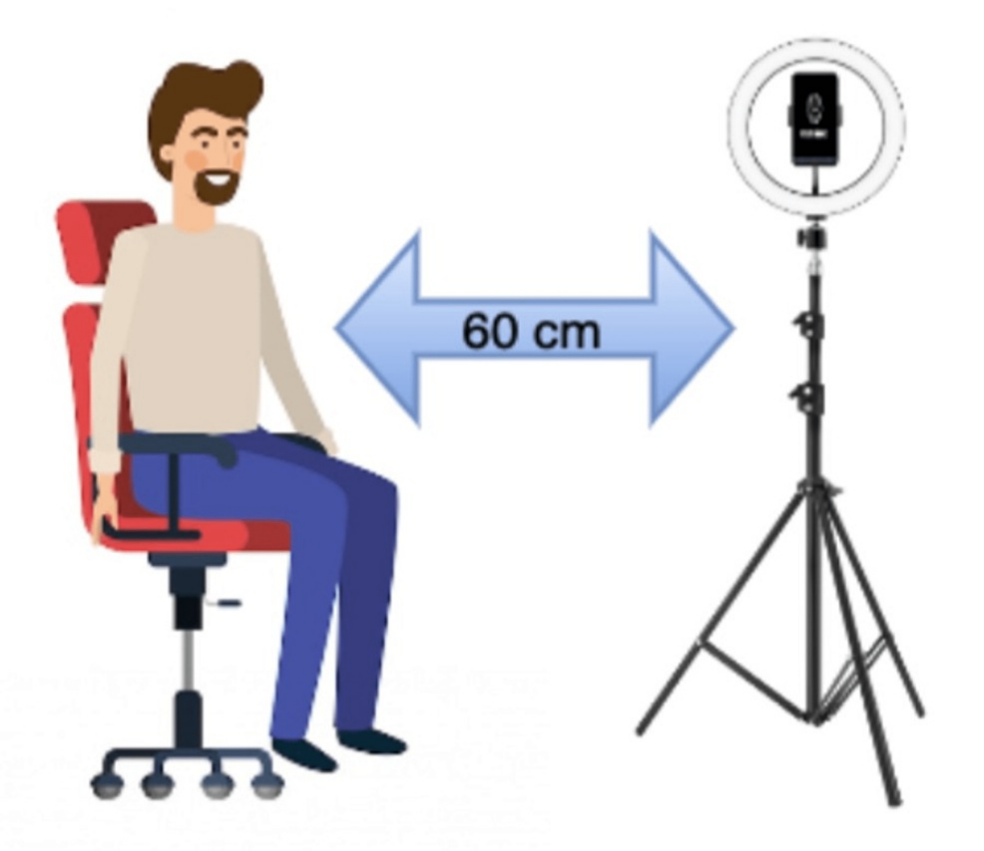
Study methodology: protocol.

Using the data collected, a pipeline was built to first parse the sample, process the BGR components into a 1D signal, and extract each one of the physiological parameters. Lastly, the results extracted were compared to the ground truth obtained with medical devices. Using the comparison between ground truth and estimated values, the metrics mean error (ME), mean absolute error (MAE), root mean squared error (RMSE), and root mean squared percentage error (RMSPE %) were calculated. Figure 3 summarizes the workflow [7].

Study design

Five vital signs, namely, HR, RR, SpO_2_, systolic blood pressure (SBP), and diastolic blood pressure (DB), were simultaneously measured using medical devices. In the study, multiple devices were used: an HP laptop with a Logitech webcam and smartphones (Samsung M32, One Plus 9, iPhone 11, and an iPad 9). The idea was to diversify the device to show that the results do not suffer from any technology bias, working in Android, iOS, Windows, and macOS systems [8].

The device was positioned 60 cm away from the subject’s face and fixed to a tripod with a ring light source in front of the subject (minimum of 250 lux). Reference measurements were extracted with Massimo mightiest for HR and RR; OMRON/CIRCA MICRO LIFE/NIDEK multi-parameter monitor and pulse oximeters. Subjects were asked to remove glasses or any other facial coverings and to be seated in a chair. During the scan, subjects were instructed to remain completely still and look straight at the camera during the entire duration of the scan. Each scan took 60 seconds and all the vitals were measured once using the medical devices. Figure 1 shows the experiment setup [9].

## Results

A total of 463 subjects were recruited for the study. In total, 179 females and 287 males, aged between 18 and 83 (42) years, with various skin colors, ethnicities, and medical statuses participated in this study. A summary of patient demographic characteristics can be found in Table 1.

**Table 1:** Demographic characteristics.

Characteristics	Mean (minimum-maximum)
Age	42.07 (18-83)
Body mass index	25.11 (7-34)
Height	165.29 (147-252)
Weight	68.45 (32-103)

Aside from evaluating the technology in various patients with different demographic information, such as age range, height, and weight, it is also important to analyze subjects with different baseline parameters, such as resting pulse rate, RR, and BP. An overview of the reference values can be seen in Table 2.

**Table 2:** Vital signs ground truth.

Vital sign	Mean (minimum-maximum)
Heart rate	80.36 (51-124)
Oxygen saturation	97.57 (91-100)
Respiration rate	17.62 (8-36)
Systolic blood pressure	125.81 (82-205)
Diastolic blood pressure	80.32 (50-119)

**Figure 4:**
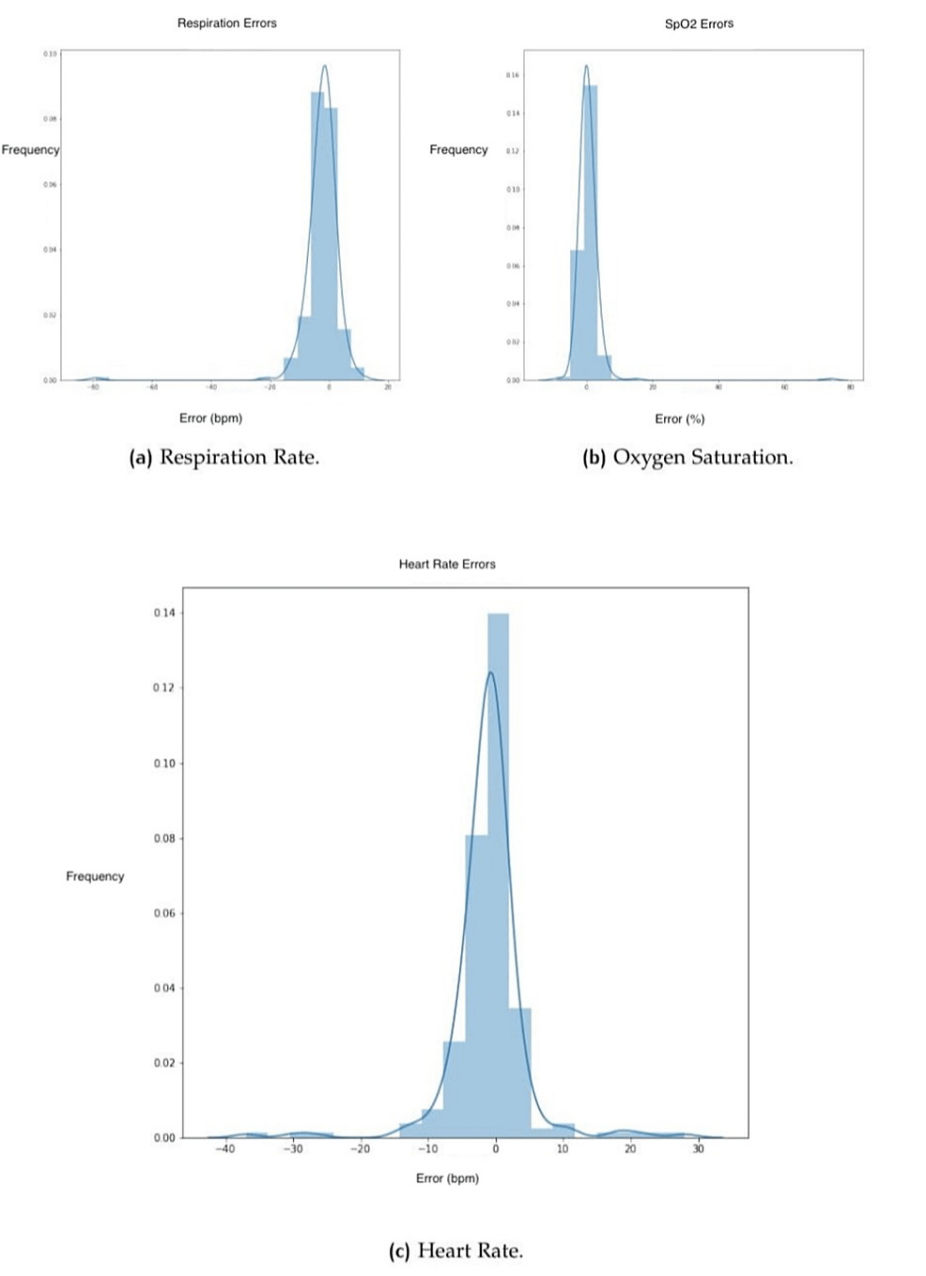
Histograms showing errors. (a) Respiration rate (RR), (b) oxygen saturation (SpO_2_), and (c) heart rate (HR).

**Figure 5:**
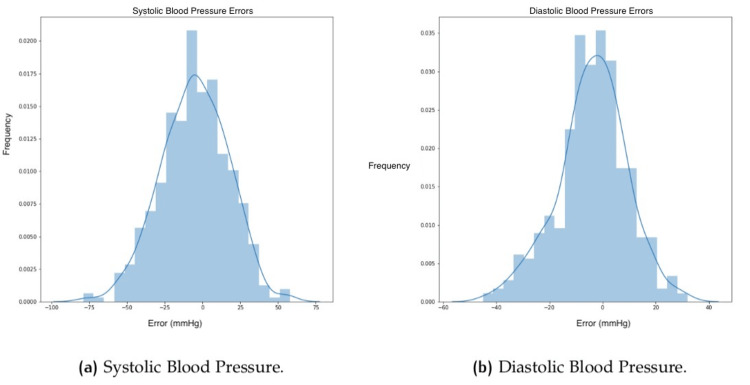
Histograms showing errors in blood pressure.

The reference values for each subject were compared to the estimated through facial scanning. Table 3 shows for each vital sign the sample size, ME, MAE, RMSE, and RMSPE. Moreover, results for each vital sign were split into three groups to present results within separate classes. Apart from the numeric values in the above table, it is also possible to evaluate the results through charts that provide a different view of the correlations. Figure 4 shows histograms of the errors for each one of the vital signs for RR, SpO_2_, and HR. Figure 5 shows the same plot for BP in terms of SBP and DBP.

**Table 3:** Table of differences between reference and estimated values. ME: mean error; MAE: mean absolute error; RMSE: root mean squared error; RMSPE: root mean squared percentage error

Vital sign	Sample size	ME	MAE	RMSE	RMSPE (%)
Heart rate (HR)	240	-1.19	3.09	5.72	7.43
HR ≤ 60	17	1.94	2.88	5.51	10.48
60 < HR ≤ 100	202	-1.17	3.01	5.31	7.12
HR > 100	21	-3.90	4	8.86	7.39
Oxygen saturation (SpO_2_)	235	0.3	1.21	1.77	1.85
90 < SpO_2_ ≤ 95	28	3.64	3.64	3.8	4.06
SpO_2_ > 95	207	-0.15	0.88	1.26	1.29
Respiratory rate (RR)	224	-2.11	3.39	4.65	25.82
RR ≤ 10	6	6.17	6.17	7.13	81.94
10 < RR ≤ 18	136	-0.48	2.02	2.7	17.94
RR > 18	82	-5.41	5.46	6.58	28.22
Systolic blood pressure (SBP)	463	-5.27	18.15	22.77	17.59
SBP ≤ 90	3	38.33	38.33	38.48	45.62
90 < SBP ≤ 130	297	3.55	14.35	17.6	15.59
SBP > 130	163	-22.13	24.69	29.68	19.93
Diastolic blood pressure (DBP)	463	-3.95	10.49	13.71	17.38
DBP ≤ 60	17	20.29	20.29	22.13	41.18
60 < DBP ≤ 90	381	-2.17	8.31	10.76	14.05
DBP > 90	65	-20.68	20.68	23.1	23.47

**Figure 6:**
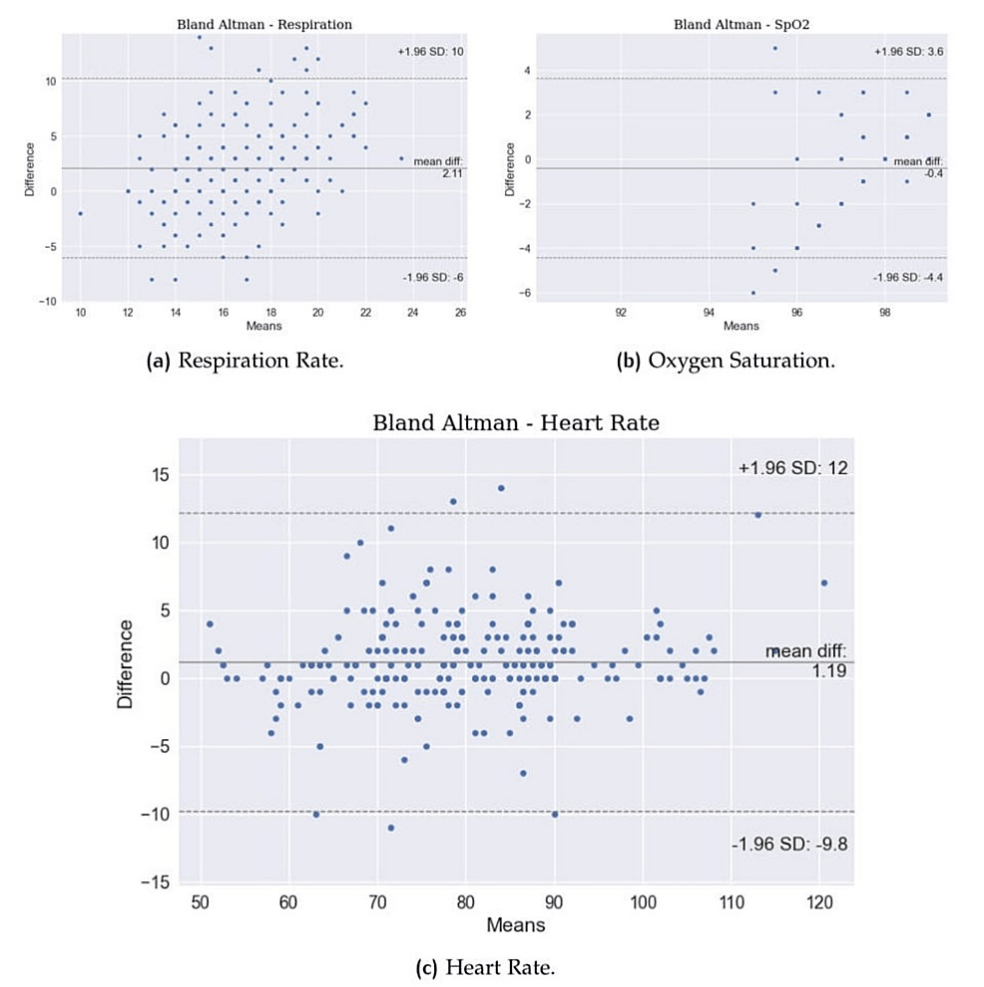
Bland-Altman plots. (a) Respiration rate, (b) oxygen saturation, and (c) heart rate.

**Figure 7:**
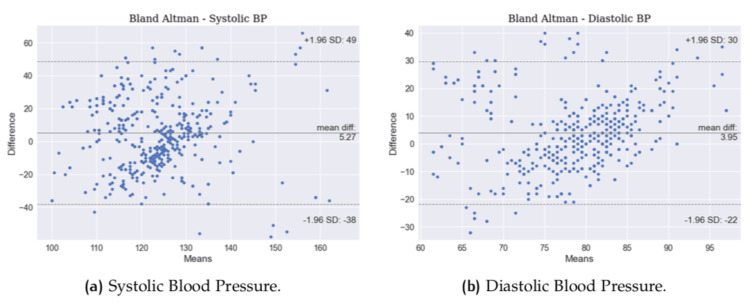
Bland-Altman plots. (a) Systolic blood pressure (SBP) and (b) diastolic blood pressure (DBP).

Despite histograms, which show the error distribution, it is also possible to evaluate through Bland-Altman plots, which show the results in terms of mean value versus difference for each data point. Figure 6 and Figure 7 show the plots for all the vitals.

 

**Figure 8:**
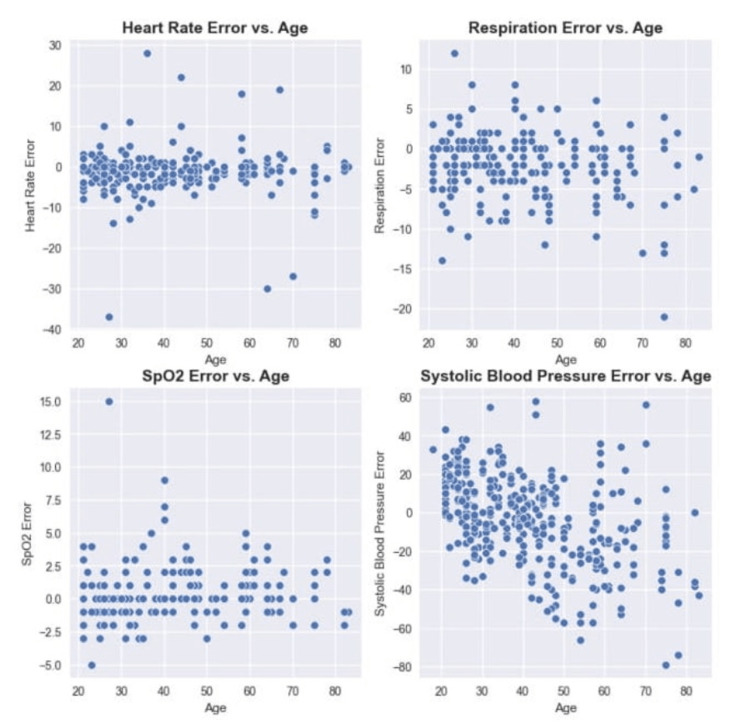
Impact of age on vitals errors for heart rate, respiration, SpO2, and systolic blood pressure.

**Figure 9:**
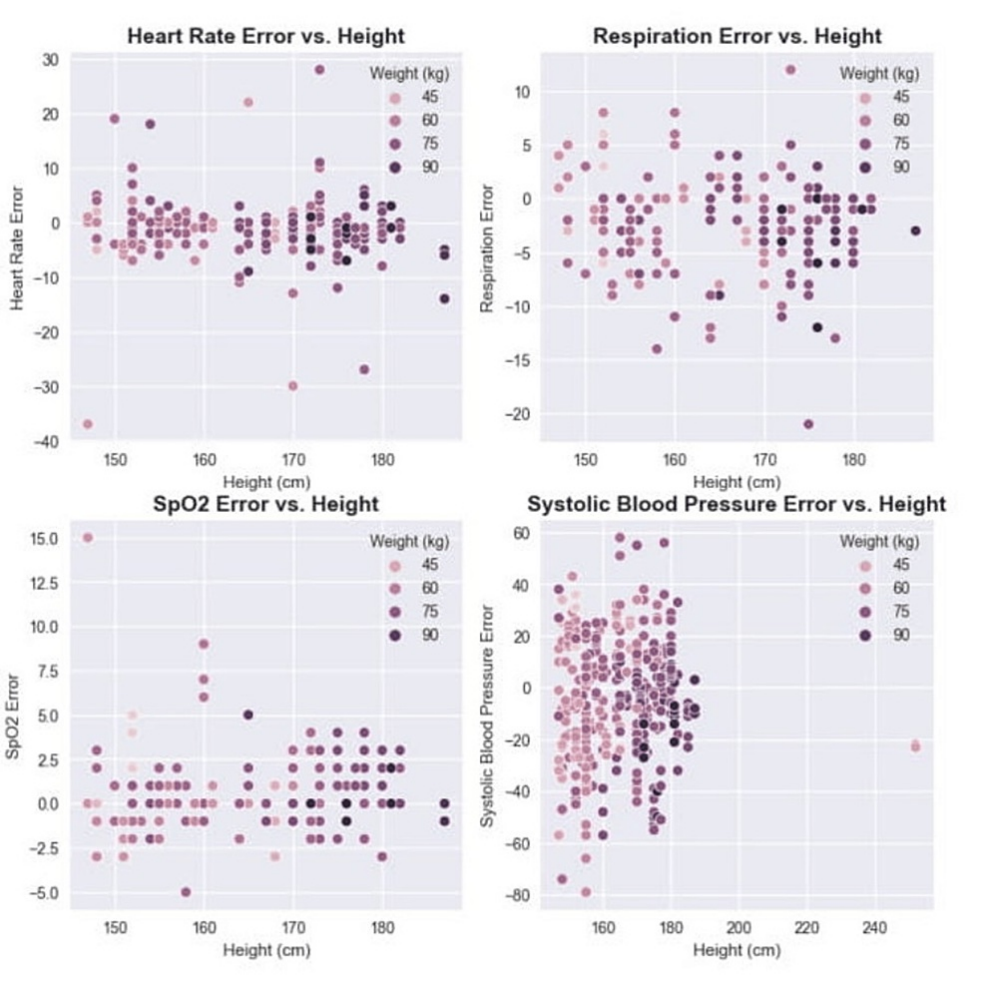
Impact of height and weight on vitals errors for heart rate, respiration, SpO2, and systolic blood pressure.

As this study proposes a deep dive into the presented results, Figure 8 evaluates the impact of age on errors. Similarly, Figure 9 shows the influence of height and weight.

**Figure 10:**
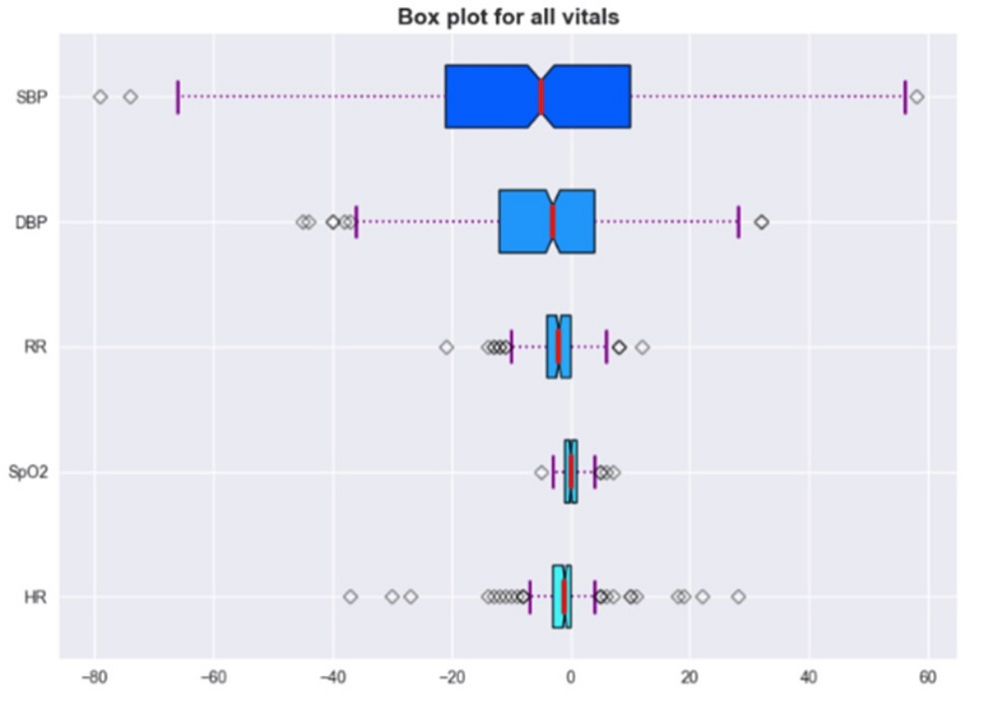
Box plot for all vitals. SBP: systolic blood pressure; DBP: diastolic blood pressure; RR: respiratory rate; SpO_2_: oxygen saturation; HR: heart rate

Furthermore, it is well known that there are going to be outliers, which might drive the statistical results away. Through box plots, this study aims to show the quartile ranges as well as the outliers for each one of the vitals, described in Figure 10.

## Discussion

This study aims to evaluate visual technology against medical devices. The technology used in this study is subject to frequent software upgrades to continuously improve its ability; therefore, the performance of the tool in studies conducted recently is likely to outperform older studies. In this study, data from 463 subjects were collected; however, not all the vitals were collected for each patient. BP is the only parameter always collected. The sample size of the others was decreased to around 240 patients [10,11].

Overall, the results show good agreement between camera-based visual monitoring technology and the reference instruments. HR, RR, and SpO_2_ reached the hypothesis criteria of ±3 units in ME for each parameter. SBP and DBP also met the requirement of ±10 units in ME; however, this should be noted with caution given the high standard deviation of error among the results [12].

With regards to the other metrics, it is possible to see that using MAE most algorithms were able to maintain similar results, apart from SBP, which highly increased in absolute difference. When applying a quadratic function, such as RMSE, it is possible to highlight errors that would be smoothed by the mean value. Having said that, RMSE from HR and SpO_2_ showed acceptable results in the average and within each group; however, in RR and further in SBP and DBP, it is possible to see the impact of large errors in a quadratic metric [13].

One of the most robust algorithms is indeed the HR estimation, which was able to maintain ±3 criteria in the average as well as within low and normal ranges. Naturally, the extreme cases of either high or low HR imply more difficulty in estimation, especially higher values that can be associated with motion artifacts involving voluntary or involuntary movement during image acquisition due to the stress and anxiety that the patients might face for obvious reasons of being at a hospital or just for participating in the research study.

SpO_2_ showed acceptable agreement between 90% and 100%, with a 0.3% ME. In this study, there were limited samples with SpO_2_ below 90% because these cases usually only occur for individuals in either a critical situation or a high altitude setting, and future work will look into including more participants with hypoxia to validate the agreement at such low levels. Nevertheless, it is worth mentioning the current scope of the technology which will more likely be used to prevent such conditions rather than be used during it [14].

RR, on the other hand, has shown the best performance in the normal respiration range between 10 and 18 breaths per minute, with a mean error of -0.48. The highest bias can be observed in very low levels of less than 10 breaths per minute.

Moreover, BP, in terms of SBP and DBP, is the most challenging problem to solve. In this study, the technology used showed good results, considering the results and the number of samples within each group; however, struggling with extreme values, mostly in SBP higher than 150 mmHg and DBP higher than 90 mmHg [15].

Considering the histograms (Figures 4, 5), it is possible to see the tendency of narrowing the plot close to error around 0, which shows that the most frequent results have lower levels of error. However, outliers might impact the statistical results. The HR histogram (Figure 4c) shows one perfect example with the classic normal (bell pattern) distribution [16].

Bland-Altman plots provide a more robust view than linear scatter plots because it is possible to see the results in terms of mean value vs. difference. Figure 6c shows that the mean difference for HR is 1.19 beats per minute, with a higher standard deviation of +12 -10, while RR shows a lower standard deviation with a higher mean difference, but with more sparse errors. SpO_2_, as should be, displays fewer data points because more than one reading had the same behavior (estimated and reference), which leads to a lower mean difference because the results are grouped. Bland-Altman plots for BP show more consistently a pattern of the wrong prediction in extreme cases with SBP lower than 120 mmHg and higher than 140 mmHg, as well as DBP lower than 75 mmHg and higher than 85 mmHg. These results show the expected behavior for this version of BP, which can generalize very well within a normal range of BP and struggle with extreme cases, both low and very high values [17].

To investigate whether age has a strong role in the error, this study has shown no correlation between age and error in any one of the vitals (Figure 8). This is meaningful once no age bias is present and can be used by patients in any part of their lives. Moreover, neither height nor weight seemed to influence the results which shows that the relationship between body weight and height (body mass index) has no visible effect in the presented results (Figure 9). Nevertheless, the results show that it does not suffer from bias related to specific groups of demographic characteristics, such as age, gender, height, or weight [18].

A box plot showed the errors in terms of their distribution into quartiles as well as the outliers. As can be seen in Figure 10, HR, RR, and SpO_2_ show low interquartile ranges, while this range increases in DBP and further in SBP. These results met the initial observations that BP has a higher standard deviation compared to the other vitals. In addition, it is also possible to see some outliers in HR estimation which can drive the statistical results and may be related to wrong ground truth measurement. Yet, HR was able to achieve acceptable results even though these outliers might be present [19].

## Conclusions

In this study, 463 readings were used to analyze the accuracy of visual technology. The study was conducted in India at multiple facilities as well as in the United Kingdom at Leeds Teaching Hospital Trust with patients with varied demographic characteristics.

The study has shown that this technology met acceptable agreement levels for mean error for HR, RR, SpO_2_, and BP; however, large deviations were apparent across several BP readings. This shows that the use of camera-based monitoring solutions is acceptable for users who want to understand their general health and wellness.
